# Narrative Review of Heart Failure Related to Cocaine Consumption and Its Therapeutic Management

**DOI:** 10.3390/jcm13237275

**Published:** 2024-11-29

**Authors:** Catléya Alawoè, Nicolas Chapet, François Roubille, Hélène Peyrière, Céline Eiden

**Affiliations:** 1 Addictovigilance Centre, Montpellier University Hospital, 34295 Montpellier, France; catalawoe@gmail.com (C.A.); c-eiden@chu-montpellier.fr (C.E.); 2 Cardiology Department, Montpellier University Hospital, 34295 Montpellier, France; n-chapet@chu-montpellier.fr; 3 Cardiology Intensive Care Department, PhyMedExp, INSERM U1046, CNRS UMR 9214, Montpellier Faculty of Medicine, Montpellier University Hospital, 34295 Montpellier, France; 4 Addictovigilance Centre, INSERM U1058, Pathogenesis and Control of Chronic Infections (PCCI), Montpellier Faculty of Pharmacy, Montpellier University Hospital, 34295 Montpellier, France; h-peyriere@chu-montpellier.fr

**Keywords:** heart failure, cocaine, beta-blockers, review

## Abstract

**Background:** Cocaine use can cause multiple cardiovascular complications, including heart failure. **Aim:** This general review of the literature delivers data on the relationship between cocaine consumption and the development of heart failure, as well as the elements of its diagnosis and management. **Methods:** A literature search was carried out using the PubMed, Web Of Science, and Google Scholar bibliographic databases over the period of 2007–2022 using the following keywords: “cocaine” AND “heart failure” NOT “acute heart disease”. The exclusion criteria exempted studies carried out on animals, along with articles not written in English. **Results and Discussion:** A total of 27 articles (11 reviews, 10 clinical studies, 4 letters to the editor, and 2 clinical cases) were included. The prevalence of heart failure among cocaine users varies from one study to another (2.5%, 5.3%, 6.2%, or even 20%); however, when patients have a history of cocaine consumption, the prevalence of heart failure is higher than that ordinarily found in the young population (<0.1% to 0.5%). Cocaine consumption has a number of serious cardiotoxic effects that can lead to heart failure. According to the studies analysed, heart failure should be treated with beta-blockers, even in the event of long-term cocaine use, with a preference for carvedilol. **Conclusions:** Despite previous concerns about the use of beta-blockers in cocaine users, treatment with beta-blockers (particularly carvedilol) may actually result in measurable clinical improvement. Cocaine withdrawal remains essential for optimal treatment.

## 1. Introduction

In France, cocaine use at least once during the year has risen among adults (aged 18–64) over the past 20 years, climbing from 0.3% in 2000 to 1.6% in 2017 [1]. This increase is more marked among adults aged over 25 years.

The French Addictovigilance Network (FAN) has reported an increase in serious cases of complications related to the use of all forms of cocaine since 2010 [2]. In 2021, 16% of spontaneous reports (SRs) to the network concerned this substance. Analysis of these SRs reveals psychiatric and somatic complications, specifically cardiovascular complications, the main instances being arterial hypertension, myocardial infarction, and heart rhythm disorders.

Rarely reported to the FAN, heart failure can be a consequence of cocaine use through direct toxicity to cardiac cells and/or indirect toxicity by increasing the heart rate and blood pressure. However, the precise clinical, physiological, and anatomical features of this disease are currently rarely documented. Despite numerous reviews examining the correlation between cocaine and acute coronary syndrome, to our knowledge, there are limited data, particularly meta-analyses, linking cocaine to heart failure.

The aim of this article was to review the existing data on the link between cocaine consumption and the development of heart failure, as well to examine the clinical challenges involved in the treatment of these patients by analysing the various articles included.

## 2. Materials and Methods

### 2.1. Summary of Inclusion Stages

The review of the literature was carried out in accordance with the recommendations of the PRISMA method (Preferred Reporting Items of Systematic Reviews and Meta-Analyses). A protocol was registered on 21 April 2023 in PROSPERO under number CRD42023417254.

A documentary search was carried out using the PubMed, Web Of Science, and Google Scholar bibliographic databases over the period of 2007–2022 with the keywords: “cocaine” AND “heart failure” NOT “acute heart disease”. The following advanced query was used in each of the search sites: “cocaine” AND “heart failure” NOT “acute heart disease”.

**Figure 1:**
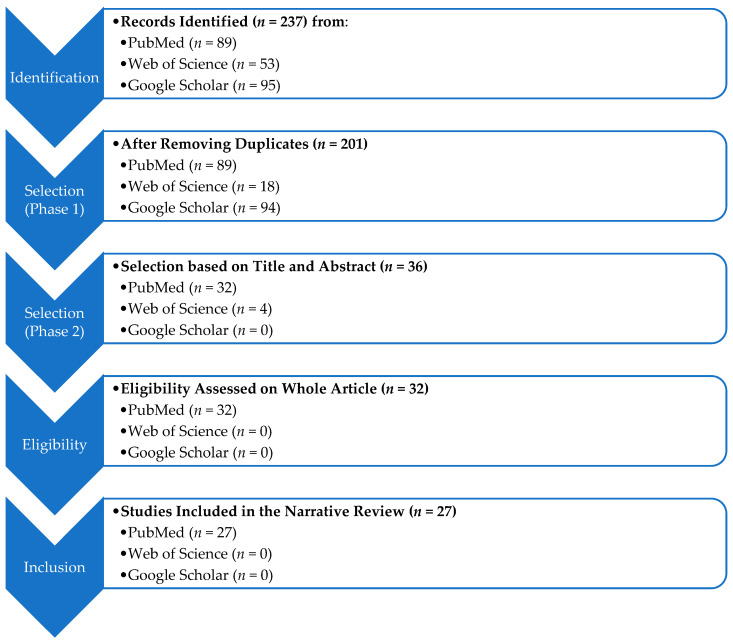
Flowchart of the study.

This search generated 89 articles on PubMed, 53 on the Web of Science, and 95 on Google Scholar. The articles were then extracted using Zotero reference management software. The PubMed database was used as a reference base in order to deduplicate the articles. A total of 35 duplicates were identified. After deleting duplicates, an initial selection of articles was conducted on the basis of the title and the abstract (selection of *n* = 36 articles). A second selection stage was carried out after reading the article with regard to exclusion criteria (inclusion of *n* = 27 articles; flowchart in Figure 1).

### 2.2. Exclusion Criteria

Articles not in English were excluded, as were experimental studies (in vitro or ex vivo) performed on animals.

## 3. Results and Discussion

The final selection included 11 reviews, 10 clinical studies, 4 letters to the editor, and 2 clinical cases (Table 1). Among these articles, the origin of the authors is European, Japanese, Austrian, and Canadian, with one case for each, and American for the others. Most of the clinical studies reviewed were retrospective, some were single-centre, and they often exhibited small sample sizes and were non-randomised. The various data from these articles have been classified according to four main highlighted themes:

- Epidemiology of heart failure and cocaine consumption;
- Pathophysiological hypotheses for the development of heart failure in cocaine consumers;
- Therapeutic management:
- Follow-up and long-term consequences.

**Table 1:** Articles included in the narrative review (n.r.: not relevant).

Article Title	1st Author/Year	Patients (*n*)	Article Type
Long Term Effects of Cocaine on the Heart Assessed by Cardiovascular Magnetic Resonance at 3T	Maceira et al., 2014 [3]	132	Cohort Study Prospective
Comparison of Frequency of Cardiovascular Events and Mortality in Patients With Heart Failure Using Versus Not Using Cocaine	Nguyen et al., 2017 [4]	267	Cohort Study Retrospective
Improvement in Clinical Outcomes of Patients with Heart Failure and Active Cocaine Use after β-Blocker Therapy	Lopez et al., 2018 [5]	38	Cohort Study Retrospective
Effect of β-Blocker Therapy on Hospital Readmission and Mortality in Heart Failure Patients With Concurrent Cocaine Use	Egbuche et al., 2018 [6]	268	Cohort Study Retrospective
Carvedilol Among Patients With Heart Failure With a Cocaine-Use Disorder	Banerji et al., 2019 [7]	2578	Cohort Study Retrospective
Clinical Outcomes of B-Blocker Therapy in Cocaine-Associated Heart Failure	Lopez et al., 2019 [8]	72	Cohort Study Retrospective
Detection of Subclinical Myocardial Dysfunction in Cocaine Addicts with Feature Tracking Cardiovascular Magnetic Resonance	Maceira et al., 2020 [9]	124	Cohort Study Prospective
Outcomes in Patients With Heart Failure Using Cocaine	Garry et al., 2022 [10]	3131	Cohort Study Retrospective
Effect of Cocaine, Amphetamine, and Cannabis Use Disorders on 30-day Readmissions of Patients with Heart Failure	Thyagaturu et al., 2022 [11]	978,217	Cohort Study Retrospective
Heavy Burden of Toxic Dilated Cardiomyopathy Among Young Adults: A Retrospective Study and Review of the Literature	Cinq-Mars et al., 2022 [12]	553	Cohort Study Retrospective
Successful Treatment of Cocaine-Induced Cardiotoxicity with Carvedilol Therapy	Öcal et al., 2015 [13]	1	Case Report
Severe Heart Failure in a Young Cocaine User	Bekki et al., 2021 [14]	1	Case Report
Cocaine Cardiotoxicity: A Review of the Pathophysiology, Pathology, and Treatment Options	Phillips et al., 2009 [15]	n.r.	Review
Alcoholic and Cocaine-Associated Cardiomyopathies	Awtry et al., 2010 [16]	n.r.	Review
Influence of Mitochondrion-Toxic Agents on the Cardiovascular System	Finsterer et al., 2013 [17]	n.r.	Review
Drug-induced Mitochondrial Dysfunction and Cardiotoxicity	Varga et al., 2015 [18]	n.r.	Review
Mechanisms of Cardiotoxicity and the Development of Heart Failure	Lee et al., 2015 [19]	n.r.	Review
Comprehensive Review of Cardiovascular Toxicity of Drugs and Related Agents	Mladěnka et al., 2018 [20]	n.r.	Review
Epidemiology of Cardiovascular Disease in Young Individuals	Andersson et al., 2018 [21]	n.r.	Review
Cocaine, Heart Failure, and Carvedilol: Triangulating the Safety of β-Blocker Therapy	Page et al., 2019 [22]	n.r.	Review
Beta-Blocker Therapy in Heart Failure Patients with Active Cocaine Use: A Systematic Review	Mann et al., 2020 [23]	n.r.	Review
Drugs of Abuse and Heart Failure	Grubb et al., 2021 [24]	n.r.	Review
Early-Onset Cardiovascular Disease From Cocaine, Amphetamines, Alcohol, and Marijuana	O’Keefe et al., 2022 [25]	n.r.	Review
Beta-Blocker Treatment of Heart Failure Patients with Ongoing Cocaine Use	Littmann et al., 2013 [26]	n.r.	Letter to the Editor
Safety and Efficacy of Beta-Blockers in Cocaine-Using Patients With Heart Failure	Richards et al., 2018 [27]	n.r.	Letter to the Editor
Beta-Blockers: A Real Antidote for Cocaine-Related Heart Disease?	Barison et al., 2019 [28]	n.r.	Letter to the Editor
Βeta-Blocker Therapy in Cocaine-Associated Heart Failure: Attempting to Mitigate the Risk	Schwier et al., 2019 [29]	n.r.	Letter to the Editor

### 3.1. Epidemiology of Heart Failure and Cocaine Consumption

The review by Andersson et al. in 2017 [21] mentioned that the incidence and prevalence of heart failure are low overall among young individuals (18–34 years), and incidence rates range from 0.02 to as high as 1.0 per 1000 person-years (prevalence: <0.1% to 0.5%), probably due to varying disease diagnoses, ascertainment criteria, diverse data sources, and geographical variation. Even if causes of heart failure in this population include substance abuse, especially cocaine use, no figures are available.

Barison et al. in 2019 [28] pointed out that cocaine is the second most widely consumed illicit drug after cannabis in Europe, affecting 3.9% of adults aged between 15 and 64 or around 13 million Europeans. Thyagaturu et al. in 2022 [11] cited data from the National Survey on Drug Use and Health, which estimates that in the previous year, around 19.3 million American adults aged over 18 consumed substances, 2/5 of them (38.5% or 7.4 million) using illicit substances such as cocaine.

In their editorial, Page et al. in 2019 [22] noted that cocaine abuse frequently coexists with heart failure. The authors cited Medicare data from a national sample of American patients hospitalised in 2014: out of 989,080 hospitalisations for heart failure, 6.2% of users (or 61,510 cases) had a documented substance use disorder, and cocaine was the most frequently associated substance (11,700 cases).

O’Keefe et al. in 2022 [25] indicated that substance consumption contributes significantly to the early onset of cardiovascular disease, and that the concept of toxic cardiomyopathy is recent and remains an under-diagnosed aetiology. The authors referred to a 2008 multicentre retrospective cohort study [30] including 11,258 patients: 594 (5.3%) of these patients had heart failure and reported having consumed stimulants (96% cocaine, 5% methamphetamine). The median age of patients with heart failure who used stimulants was 49.7 years compared with 76.1 years for those who did not. A comparison of the two groups showed that the proportion of patients with reduced left ventricular ejection fraction (rLVEF) was 76% for stimulant users versus 49% for non-stimulant users (OR 3.4 CI_95%_ 2.8–4.2). Stimulant users were more likely to have multiples hospitalizations (≥3) within the last six months (28% versus 11%).

Cinq-Mars et al. in 2022 [12] presented the results of their retrospective cohort study of 553 patients under 65 years of age with heart rLVEF followed-up between 2003 and 2019. Of the 201 patients (36%) with idiopathic aetiology, substance abuse could be suspected in 38 (19%) (amphetamines (50%, *n* = 19), cocaine (37%, *n* = 14), anabolic steroids (8%, *n* = 3), and energy drinks (5%, *n* = 2)). Patients in this subgroup (*n* = 38) were predominantly male (92%) and of a young average age of 40 ± 9 years (71% under 45).

Finally, even if the consumption of recreational substances is a risk factor for cardiovascular events, particularly heart failure, its exact prevalence among patients has not been established. In the various articles included, depending on the population studied (general, cardiology), the date of publication, and the method used to identify cocaine use (declarative, analytical), the figures vary, i.e., 2.5%, 5.3%, 6.2%, or even 20%.

In France, a recent multicentre prospective study [31] was carried out on patients admitted to cardiac intensive care units. The prevalence of recreational substance use was 11% in this sector, with cocaine in third place after cannabis and opioids.

### 3.2. Pathophysiological Hypotheses for the Development of Heart Failure in Cocaine Users

In their 2021 review, Grubb et al. [24] detailed the direct electrophysiological/molecular effects of cocaine on the myocardium, e.g., inhibition of L-type calcium channels, KCNH2-type potassium channels, and voltage-dependent sodium channels, which cause abnormalities in myocyte function (hypertrophy, altered calcium regulation, inflammation, fibrosis, reduced lusitropy, and inotropy), leading to difficulty in cardiac propulsion.

Phillips et al. in 2009 [15] and Varga et al. in 2015 [18] indicated that the significant release of reactive oxygen species (activation of NADPH oxidase, dysregulation of xanthine oxidase, auto-oxidation of excess catecholamines) following cocaine consumption may also be associated with cardiomyocyte apoptosis and therefore, with the progression of heart failure.

Finally, Finsterer et al. in 2013 [17] reported that around 45% of myocardial volume is occupied by mitochondria. Impaired mitochondrial function leads to the death of cardiomyocytes and endothelial cells, resulting in cardiovascular dysfunction. Cocaine is known to have a direct or indirect effect on mitochondrial function, particularly in the heart.

According to Varga et al. [18], Lee et al. [19], Phillipes et al. [15], and Grubbs et al. [24], cocaine causes vascular pathophysiological effects (increase in endothelin-1, decrease in nitric oxide, vasoconstriction, and vasospasm) and thrombotic effects (increase in platelet activity, thrombosis, and accelerated atherosclerosis).

Awtry et al. in 2010 [16] stated that the pathophysiological findings of cocaine-induced cardiomyopathy are generally similar to those observed in patients with other forms of heart failure, with a few exceptions, i.e., more acute onset, with less common lower limb oedema as a sign of chronic heart failure. Furthermore, compared with reports of myocardial inflammation in 1–9% of cases studied during routine autopsies, 20% of patients who died with a positive cocaine screen showed signs of myocarditis. Bekki et al. [14] in 2021 reported the case of a 27-year-old man who presented with severe heart failure induced by lymphocytic myocarditis, probably caused by cocaine consumption.

According to Phillips et al. [15] and O’Keefe et al. [25], specifically, the increased myocardial sympathetic activation in cocaine users is probably an attempt at autonomic compensation for reduced cardiac output. This over-stimulation of the adrenergic system leads to damage such as increased cardiac hypertrophy, decreased left ventricular end-diastolic volume, and increased wall thickness. These changes have been observed during electrocardiographies of cocaine-addicted patients, which show abnormal increases in the amplitude and duration of R waves, shifts of the J point (the point where the QRS wave ends and the ST segment begins) to the right, and changes in the frontal leads (I, II, aVL, aVR, aVF) on ECGs compatible with left ventricular hypertrophy.

In 2014, Maceira et al. [3] used cardiac MRI to confirm the cardiac effects of chronic cocaine consumption in an initial prospective study. In 94 adults (aged 18 to 60) with cocaine dependence matched to a control group, imaging revealed, in addition to the changes already mentioned, an increase in gadolinium uptake in the heart muscle, indicating damage to the heart. In 2020 [9], a second prospective study assessed cardiac parameters using nuclear magnetic resonance (NMR) and Nuclear Magnetic Resonance with tissue deformation analysis (NMR-FT) in three groups of patients: 62 patients with cocaine use disorder and preserved left ventricular ejection fraction (pLVEF) versus 32 patients with cocaine use disorder and rLVEF versus 30 controls. Patients with cocaine use disorder appear to develop predominantly rLVEF rather than preserved or intermediate heart failure, with significant alterations in cardiac structure, function, and deformity; however, there is a lack of specific studies on this topic that take into account confounding factors (e.g., smoking). At autopsy, remodelling resulted in myocardial weight that was on average 10% higher than expected. Knowledge of these pathophysiological mechanisms is of interest as a basis for research into new therapeutic targets.

Among the confounding factors, tobacco and alcohol are often considered frequent causes of cardiomyopathy. In the studies analysed, active smoking was the most common comorbidity among cocaine users (up to 87% of subjects [12]). With regard to smoking, a meta-analysis in 2019 and a clinical study in 2022 including 9345 subjects aged between 61 and 81 showed its association with the risk of heart failure [32,33]. For alcohol and heart failure, the data do not appear to be as conclusive [34].

### 3.3. Therapeutic Management

The American College of Cardiology/American Heart Association recommends initiating treatment with beta-blocker drugs as soon as left ventricular dysfunction is diagnosed [35]. These medicines have been shown to be effective in alleviating the symptoms of heart failure, while reducing the risk of hospitalisation and improving survival.

However, Barison et al. in 2019 [28] repeated that the debate concerning the use of beta-blockers in cocaine-using patients stems from the publication of clinical cases in 1985 and 2007. Indeed, for patients with acute decompensated heart failure who were cocaine users, metoprolol and labetalol did not provide optimal therapeutic management. Based on this clinical finding, the authors suggested that cocaine consumption, which increases the release of catecholamines and over-stimulates the alpha-adrenergic receptors, combined with a ß1-cardioselective beta-adrenergic blocking agent, would lead to coronary vasoconstriction and paradoxical hypertension due to the lack of opposition to the stimulation of alpha-adrenergic receptors, therefore advising against the use of beta-adrenergic blocking agents in these patients. With regard to non-cardioselective beta-blockers contraindicated in spastic angina, the initial fear regarding cocaine users was the risk of vasospasm on introduction of these treatments, thus discouraging the use of beta-blockers in these patients.

Schwier et al. in 2019 [29], in response Barison et al. [28], proposed a beta-blocker-free therapeutic alternative following the results of the RALES clinical study, e.g., the combination of an angiotensin-converting enzyme inhibitor and an aldosterone receptor antagonist, such as spironolactone, described as having significantly reduced the risk of all-cause death in heart failure patients with rLVEF, even in the absence of beta-blockers. However, this possibility only applies to patients with symptoms of NYHA class III-IV heart failure and a LVEF ≤ 35% (RALES criteria), as reiterated in the 2016 ESC recommendations. According to the latest ESC recommendations, aldosterone antagonists should be initiated in patients with rEVF (grade Ia) and moderately impaired LVEF (grade IIa).

In 2013, Littmann et al. [26] reported the cases of four patients with severe heart failure and active cocaine consumption. These patients were treated with carvedilol (a non-cardioselective beta-blocker) at a target dose of 25 mg twice daily for a period of two years. Despite active cocaine consumption, none of the patients required rehospitalisation to the cardiology department within a year, and the authors noted clinical improvement and almost complete recovery of their LVEF.

In 2015, Öcal et al. [13] published a case report of a 34-year-old patient who developed acute myocarditis with rLEVF following cocaine consumption. Treatment consisted of a combination of aspirin, 100 mg/day, and carvedilol, 6.25 mg twice daily. After one week of treatment, the clinical and echocardiographic evaluations were normal.

Nguyen et al. in 2017 [4] carried out a single-centre retrospective study between January 1993 and May 2012 on patients hospitalised for acute decompensated heart failure by comparing the frequency of major adverse cardiovascular events and mortality in cocaine-consuming patients (*n* = 90) versus non-consumers (*n* = 177). Among patients with heart failure and cocaine consumption, 71% had been treated with beta-blockers, 60% of them with cardioselective and 40% with non-cardioselective types. Patients with heart failure who consumed cocaine had a better prognosis using beta-blockers. The authors stressed the importance of taking into account the quantity of cocaine consumed by patients, since higher quantities could increase the risk of adverse effects linked to the activation of alpha-adrenergic receptors.

Egbuche et al. in 2018 [6] carried out a retrospective monocentric study between January 2011 and December 2014 on the effect of beta-blockers in terms of readmission and mortality in a cohort of patients hospitalised for heart failure (*n* = 6025) exhibiting active cocaine use (*n* = 325, 5%) and rLVEF (study population *n* = 268). A total of 86% of patients had been prescribed a beta-blocker for at least 1 year (beta-blocker, *n* = 231, versus no beta-blocker, *n* = 37). The authors observed a 5.9-fold reduction in the 30-day heart failure readmission rate, a 5.3-fold drop in the all-cause mortality, and a decrease in the all-cause mortality rate at 1 year in the beta-blocker group versus the no beta-blocker group.

Lopez et al. published two retrospective single-centre cohort studies in 2018 [5] and 2019 [8]. The first [5] was conducted between January 2010 and June 2016 in patients with rLVEF heart failure and active cocaine consumption treated with beta-blockers for 1 year (*n* = 38). A total of 60% of patients were treated with non-cardioselective beta-blockers versus 40% with cardioselective beta-blockers. After one year on beta-blockers, the authors observed a significant improvement in cardiac symptoms (improvement in NYHA stage and improvement in LVEF) in these patients.

The second study [8] compared this same population (*n* = 38) to a control group (*n* = 34) of heart failure patients with rLVEF and those who were active cocaine consumers, without beta-blockers. After one year, the improvement in cardiac symptoms was significantly greater in patients treated with beta-blockers compared to those without, with carvedilol showing a significant improvement in NYHA stage (OR 7.86; CI_95%_ 2.20–32.89; *p* = 0.0012) and an enhancement in LVEF (RR 2.59; CI_95%_ 1.30–5.15; *p* = 0.0051) at 12 months compared with the results of no treatment.

In 2019, Banerji et al. [7] published a retrospective single-centre cohort study conducted in 2011 on the efficacy/tolerability of carvedilol in patients managed for acute decompensated heart failure (*n* = 2578) with cocaine consumption (study population *n* = 503, 20%). Of the 503 patients, 404 (80%) were treated with carvedilol, and 99 (20%) were not treated with any beta-blockers. The results show that the cardiovascular mortality and 30-day readmission rates for heart failure are similar between the two groups. However, after stratification by stage of heart failure, the use of carvedilol was associated with a lower rate of mortality and readmission for heart failure at 30 days in patients with rLVEF. In addition, the use of carvedilol in this cohort was also associated with lower NT-proBNP levels at the time of admission and upon discharge from hospital, suggesting a cardioprotective effect of carvedilol in this population.

Finally, the systematic review by Mann et al. published in 2020 [23] examined the effect of beta-blockers in patients with heart failure who were also consuming cocaine. The authors pointed out that there were no clear treatment recommendations for these patients. Analysis of the twelve articles from 2010 to 2019 (including those by Littmann et al. [26], Ocal et al. [13], Nguyen et al. [4], Egbuche et al. [6], and Lopez et al. [5,8]) on the subject shows that the use of beta-blockers in patients suffering from heart failure who are also active cocaine consumers seems safe and beneficial, although the evidence is not very solid due to a lack of large-scale studies.

Currently, the ESC Guidelines 2021 recommend quadritherapy in the management of rLVEF (ACE inhibitors or RNAi angiotensin-neprilysin receptor blockers, fixed-combination sacubitril/valsartan (Entresto^®^), beta-blockers, mineralocorticoid receptor antagonists, and SGLT2 receptor blockers, i.e., glifozines), but make no mention of specific management in the cocaine-user population. In this context, the benefit/risk ratio of these new treatments with new pharmacological targets needs to be clarified.

### 3.4. Follow-Up and Long-Term Consequences

Thyagaturu et al. in 2022 [11] used a database of hospital admissions from 28 geographically dispersed states in the USA (representing 60% of the total population) for the years 2016 and 2018.

Among the 978,217 hospital admissions for heart failure meeting the inclusion criteria (patients aged 18 years or older with a principal diagnosis of heart failure during hospitalisation and hospitalisations occurring between 2016 and 2018), 3.5% of patients exhibited a concomitant substance use disorder (involving cocaine, other stimulants, and cannabis). Compared to patients without a substance use disorder, patients with a substance use disorder displayed a significantly higher rate of readmission for all-cause mortality within 30 days (22.6% compared with 17.6%, with an adjusted relative risk of 1.16 [1.12–1.21], *p* < 0.01).

Garry et al. in 2022 [10] published a retrospective single-centre cohort study of patients with a diagnosis of heart failure admitted between January 2001 and July 2019. Of these, 791 patients (25.3%) were cocaine consumers. After adjustment for demographic variables, comorbidities, and associated substance consumption, cocaine use was not significantly associated with an increase in all-cause mortality at 1 year (HR 1.10 CI_95%_: 0.78–1.55), 5 years (HR 1.13 CI_95%_: 0.91–1.40), and 10 years (HR 1.18 CI_95%_: 0.97–1.43). However, cocaine use significantly increased the risk of all-cause mortality over the entire follow-up period (HR 1.21 CI_95%_: 1.00–1.48), as well as the risk of 90-day readmissions. In their subgroup analysis, the prescription of beta-blockers (metoprolol or carvedilol) at the time of discharge resulted in no significant difference in terms of mortality and readmission rate compared with the rates for those who did not receive a prescription.

In the review by Grubb et al. [24], the authors pointed out that heart failure patients with a history of cocaine consumption are often younger than the general heart failure population, which may result in a more favourable prognosis for the disease. However, in the reviews by Awtry et al. [16] and Phillips et al. [15], the authors stated that it is essential to consider total cessation of cocaine consumption as a primary objective in the long-term management of these patients. In particular, the results are based on case reports describing a marked improvement in left ventricular function (return to normal cardiac dimensions) and clinical outcome (enhancement in LVEF, normalisation of systolic and diastolic functions) in patients after stopping cocaine consumption (between 5 and 9 months after cessation). In addition, it has been observed that left ventricular dysfunction and heart failure may reappear if the patient resumes consumption.

In the Cinq-Mars et al. cohort in 2022 [12], the majority of patients had a favourable outcome after stopping the consumption of stimulants, with an increase in their LVEF, with some even achieving complete recovery. However, a small proportion of patients required advanced therapies, such as mechanical circulation or heart transplantation.

**Figure 2:**
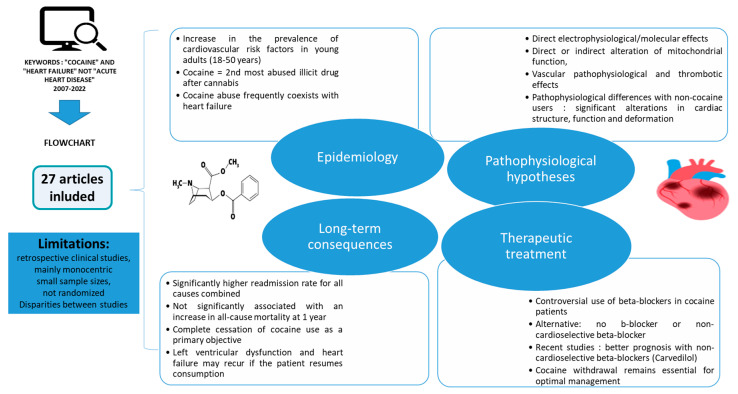
Narrative review of heart failure related to cocaine consumption and its therapeutic management.

The overall data are summarized in Figure 2. In the analysed studies, information was lacking regarding the adherence to treatment, as well as the quantity and route of cocaine consumption, information that is crucial for all studies on the management of substance use disorders. There are also confounding factors that can introduce bias and affect the validity and generalizability of the results. Moreover, subjectivity in study selection is the main weakness ascribed to narrative reviews, potentially leading to biases.

Optimal treatment of cocaine-related heart failure requires the cessation of cocaine consumption, since sustained cocaine use may continue to worsen heart problems. The effects of cocaine on the heart may be chronic and evolve over time, meaning that these data are important, but not well-documented in the literature.

According to some authors, cocaine withdrawal plays an important role in the management of patients with heart failure. However, there are no studies evaluating this or discussing the medical monitoring of withdrawal symptom management, psychological support, and/or rehabilitation, which can be particularly intense in cocaine-dependent patients, in order to maintain long-term abstinence [36].

## 4. Conclusions

This narrative review has shown that this subject has been little studied, particularly in Europe, with data coming mainly from American centres. The available data highlight the serious and potentially irreversible risks to cardiac health associated with cocaine use. Despite previous concerns about the use of beta-blockers in these patients, treatment with beta-blockers (particularly carvedilol) may actually result in measurable clinical improvement. Cocaine withdrawal remains essential for optimal treatment.

## Data Availability

No new data were created or analyzed in this study. Data sharing is not applicable to this article.
